# Coronavirus disease 2019 (COVID-19) outbreak during a Chinese New Year dinner in a restaurant, Hong Kong Special Administrative Region SAR (China), 2020

**DOI:** 10.5365/wpsar.2020.11.2.006

**Published:** 2021-02-16

**Authors:** Tsz-sum Lam, Chi-hong Wong, Wing-hang Lam, Ho-yeung Lam, Yonnie Chau-kuen Lam, Emily Chi-mei Leung, Shuk-kwan Chuang

**Affiliations:** a Communicable Disease Branch, Centre for Health Protection, Department of Health, Hong Kong SAR (China).

Coronavirus disease 2019 (COVID-19) was first detected in Wuhan, China, on 31 December 2019. (*1*) The first confirmed case of COVID-19 imported from Wuhan to  Hong Kong Special Administrative Region SAR (China) was recorded on 23 January 2020, (*2*) and on 30 January 2020 the World Health Organization (WHO) declared the COVID-19 outbreak a public health emergency of international concern. (*3*) Between January and May 2020, 1084 confirmed cases of COVID-19 were reported in Hong Kong Special Administrative Region SAR (China). The local epidemic progressed through four phases: (1) preparedness and imported infection from mainland China, (2) local transmission, (3) imported infection from overseas countries associated with local transmission, and (4) controlled imported infection with limited local transmission. (*4*) During the second phase – local transmission (4 February to 3 March) – we reported a local family cluster of six confirmed COVID-19 cases among 29 people who attended a Chinese New Year family dinner gathering in a restaurant on 26 January 2020 (the second day of Chinese New Year).

## Methods

We conducted an epidemiological investigation of a confirmed case of COVID-19. On 10 February 2020, we received notification of a confirmed case of COVID-19 involving a 37-year-old female (patient 1) who had developed fever, cough and sore throat from 2 February 2020. She was admitted to a public hospital on 10 February 2020 and her nasopharyngeal aspirate tested positive for severe acute respiratory syndrome coronavirus 2 (SARS-CoV-2) ribonucleic acid (RNA) using real-time reverse transcription polymerase chain reaction. Symptomatic contacts were isolated in public hospitals for SARS-CoV-2 testing and management. Asymptomatic close contacts were quarantined in quarantine centres, while other contacts who were asymptomatic were put under medical surveillance.

## Results

Patient 1 was home based, had no recent travel history outside Hong Kong Special Administrative Region SAR (China) and denied having any contact with confirmed COVID-19 cases. Contact tracing revealed that her husband (patient 2), who resided with her, developed fever and cough on 30 January 2020. In addition, her father (patient 3), who did not reside with her, developed fever and cough from 3 February 2020. Patients 2 and 3 were admitted for isolation on 10 February 2020 and tested positive for SARS-CoV-2.

Patients 1–3 shared a Chinese New Year dinner with 26 other relatives on 26 January 2020. Between 31 January and 8 February 2020, three more relatives were found to be symptomatic and tested positive for SARS-CoV-2 (patients 4–6).

The 29 attendees at the dinner lived in various separate residences, and the family dinner was the only common exposure among all six confirmed cases during the incubation period. The dinner, which was held in a restaurant, lasted for about 7 hours and included mahjong playing. Three of the six confirmed cases had played mahjong. The 29 diners were seated at two tables in the same room, in a partitioned area within the restaurant. None of the attendees were symptomatic during the gathering. The restaurant where the outbreak occurred was closed permanently because of business considerations before the notification of a COVID-19 case on 10 February 2020. Hence, it was not possible to undertake contact tracing of the wait staff or environmental investigations.

**Figure 1:**
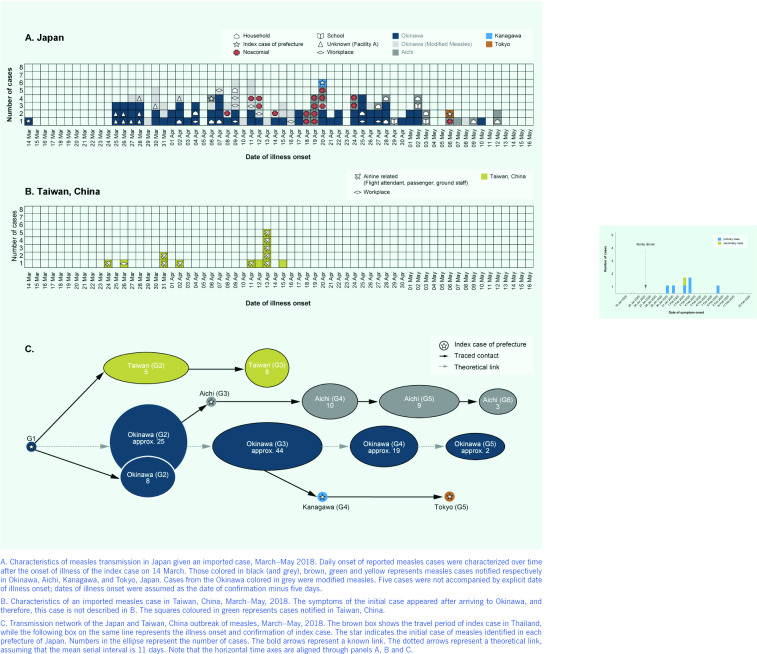
Epidemic curve of Chinese New Year restaurant dinner COVID-19 outbreak

Contact tracing identified one domestic helper (patient 7) who did not join the family dinner but shared a bedroom with patient 4 (symptom onset on 31 January); patient 7 developed a fever and cough from 2 February and tested positive for SARS-CoV-2 (**Fig. 1**) (four other members of that household were sent to a quarantine centre and were not infected).

In summary, the cluster was six (from the family cluster) plus one (the domestic helper) confirmed cases, comprising three males and four females aged between 32 and 75 years (median: 37 years). None of the seven patients had a travel history outside  Hong Kong Special Administrative Region SAR (China) and they all denied having any contact with confirmed COVID-19 cases during the incubation period; all seven were discharged home uneventfully.

[**insert** Figure 1]

## Discussion

WHO advises maintaining social distancing of at least 1 m (3 feet) as a basic protective measure against  COVID-19. (*5*) Mahjong is generally played by four people sitting around a square table in close proximity for hours, with the distance between players usually being less than 1 m (a distance at which transmission of respiratory droplets is possible). A Chinese dinner is commonly shared by 12 diners sitting close together at a round table, but in this particular instance, 29 diners were seated at two tables that usually accommodated 24 people, further reducing the distance between people.

This investigation had limitations. Information on viral load might have indicated who was more likely to be the heavier spreader, but no laboratory investigation of viral load was conducted. Also, it was not possible to undertake contact tracing of the wait staff or conduct environmental investigations.

It appears that some people can be positive for COVID-19 for 1 to 3 days before they develop symptoms. (*6*) Although the source of the family cluster could not be identified, our findings support pre-symptomatic transmission and effective human-to-human transmission of COVID-19 through social activities. Non-pharmaceutical interventions (e.g. social distancing) have been associated with reduced transmission of COVID-19 in  Hong Kong Special Administrative Region SAR (China). (*7*) The Centre for Health Protection appeals to the public to properly maintain social distancing at all times during the COVID-19 pandemic.
